# Importance of early detection of juvenile polyposis syndrome: A case report and literature review

**DOI:** 10.1097/MD.0000000000023494

**Published:** 2020-12-11

**Authors:** Na Shen, Xiong Wang, Yanjun Lu, Fang Xiao, Juan Xiao

**Affiliations:** a Department of Laboratory Medicine; b Department of Gastroenterology; c Department of Obstetrics and Gynecology, Tongji Hospital, Tongji Medical College, Huazhong University of Science and Technology, Wuhan, China.

**Keywords:** *BMPR1A*, early detection, genetic testing, juvenile polyposis syndrome, *SMAD4*

## Abstract

**Rationale:**

Juvenile polyposis syndrome (JPS) is a rare genetic gastrointestinal disorder with hidden and variable clinical features. Early detection is crucial for good prognosis.

**Patient concerns:**

A 20-year-old female went to hospital for fever, and was unexpectedly diagnosed as JPS during treatment. She reported no clinical signs or family history of JPS.

**Diagnosis:**

Blood routine examination on hospital admission suggested a moderate anemia. Bone marrow cytology and leukemia fusion gene test were performed to rule out leukemia. Other examinations including ultrasound and computed tomography were also conducted for differential diagnosis. Further electronic colonoscopy identified more than 20 pedicle polyps located at her ileocecum and rectum. Mutation analysis detected a novel de novo pathogenic variant, c.910C>T (p.Gln304Ter) within *bone morphogenetic protein receptor type 1A* gene, establishing the diagnosis of JPS.

**Interventions:**

The patient was treated with endoscopic interventions. We also provided a genetic counseling for this family.

**Outcomes:**

The patient's polyps were removed, some of which already had adenomatous changes. The patient received surveillance of hereditary colorectal cancer according to guidelines.

**Lessons:**

Variable features and lack of family history probably lead to a great underestimation of potential JPS population. It is recommended to perform genetic testing by a multigene panel in individuals who have suspected symptoms of polyposis.

## 1 Introduction

Juvenile Polyposis Syndrome (JPS, OMIM #174900) is a rare autosomal-dominant disorder, which is characterized by multiple hamartomatous juvenile polyps in the colorectum (98%), stomach (14%), duodenum (7%), and jejunum and ileum (7%).^[1]^ The incidence of JPS is between 1/100,000 and 1/160,000 individuals.^[2]^ The average age at diagnosis of JPS is 18.5 years, but solitary juvenile polyps develop at any age.^[1,3]^ JPS patients are estimated to suffer 9% to 50% lifetime risk of gastrointestinal (GI) cancers, mostly colorectal cancer (CRC). The incidence of JPS-related CRC is 17% to 22% by age 35 years and reaches 68% by age 60 years.^[4,5]^

Loss of function in *bone morphogenetic protein receptor type 1A* (*BMPR1A*) or *SMAD family member 4 (SMAD4)* gene is uncovered to be an important molecular pathogenesis of JPS. *BMPR1A* and *SMAD4* are tumor suppressor genes of TGF-β signaling pathway. Nearly 60% of JPS patients could identify causative variants in either of them.^[4]^*SMAD4* is the first gene reported to cause JPS.^[6]^*SMAD4* mutation carriers often present variable combined features of JPS and hereditary hemorrhagic telangiectasia (HHT), also called JPS/HHT syndrome.^[7]^ The clinical features of *SMAD4* -related disorders are well established.^[7]^ By contrast, most *BMPR1A*-related JPS patients are asymptomatic or have nonspecific symptoms difficultly differentiated with other GI diseases. Moreover, up to 67% of patients have no related family history, increasing more difficulty for diagnosis. To identify JPS by an early and effective way is becoming an urgent clinical issue.

Here, we reported a 20-year-old female patient who was unexpectedly diagnosed as JPS during treatment for infection. By the genetic testing of a multigene panel, we identified a novel de novo mutation of *BMPR1A* gene in this Chinese JPS family. In view of hidden and variable clinical features of JPS and insufficient review about *BMPR1A* gene, we further summarized latest evidence to better identify features of *BMPR1A*-related disorders.

### 1.1 Consent statement

This study was approved by the Ethics Committee of Tongji Hospital of Tongji Medical College of Huazhong University of Science and Technology, and was performed in accordance with the principles of Declaration of Helsinki. Written informed consents for clinical information, blood samples, and paper publication were obtained from all included subjects.

## 2 Case report

The patient was a 20-year-old female, who was investigated for fever and emesis in April, 2019. Physical and laboratory examination suggested an acute upper respiratory tract infection (body temperature: 38.8°C; white blood cell count: 1.237 × 10^10^/L, neutrophilic granulocyte count: 1.097 × 10^10^/L; detection of pathogenic IgM antibodies about respiratory tract infection: Influenza virus A (+), chlamydia pneumoniae (+), and legionella pneumophila (+)). During the treatment of infection, the patient was found to also have anemia. She considered it due to dietary habit and did not attach great importance to that. Except for that, she denied any disease history including diabetes, hypertension, or gastrointestinal diseases. She also denied family history of any genetic disease. Blood routine examination on hospital admission suggested a moderate anemia (hemoglobin: 62 g/L, mean corpuscular volume: 56.6 fL, mean corpuscular hemoglobin: 14.9 pg; and mean corpuscular hemoglobin concentration: 263 g/L). Further bone marrow cytology and leukemia fusion gene test ruled out a diagnosis of leukemia. Electronic colonoscopy identified more than 20 pedicle polyps (0.4 cm–2.5 cm) located at her ileocecum and rectum. Pathological examination after therapeutic endoscopy suggested a diagnosis of JPS, and there was villus-tubiform adenoma with high-grade dysplasia in some parts. Painless gastroscopy did not find any polyps. The patient's father also had colorectal polyps, but pathological examination demonstrated that the polyps was hyperplastic. The patient's mother reported no abnormal symptoms.

After genetic counseling, the patient and her parents underwent genetic testing. First, a next-generation sequencing (NGS) panel was applied to explore the candidate variants of the patient. The NGS panel targeted on 50 genes associated with hereditary gastrointestinal cancer syndromes including *SMAD4* and *BMPR1A* (Supplementary Table 1), which was performed using the Ion Torrent PGM platform (Thermo Fisher Scientific, Waltham, MA). The clinical significance of candidate variants was determined based on the guidelines of American College of Medical Genetics and Genomics,^[8–10]^ and analyzed by several mainstream databases, such ClinVar (https://www.ncbi.nlm.nih.gov/clinvar/) and HGMD (http://www.hgmd.cf.ac.uk/ac/index.php). Second, the identified candidate variant was further validated in this patient and her parents by Sanger sequencing using the ABI 3500 Dx Genetic Analyzer (Thermo Fischer Scientific, USA). Primers were designed to amplify the exon 10 of *BMPR1A* gene (NM_004329.2): forward, 5’-TATCCAGAATGAGCATTACTTCTCC-3’; and reverse, 5’- ATTCCATTATCTTGAATACAAATAGGG-3’.

**Figure 1:**
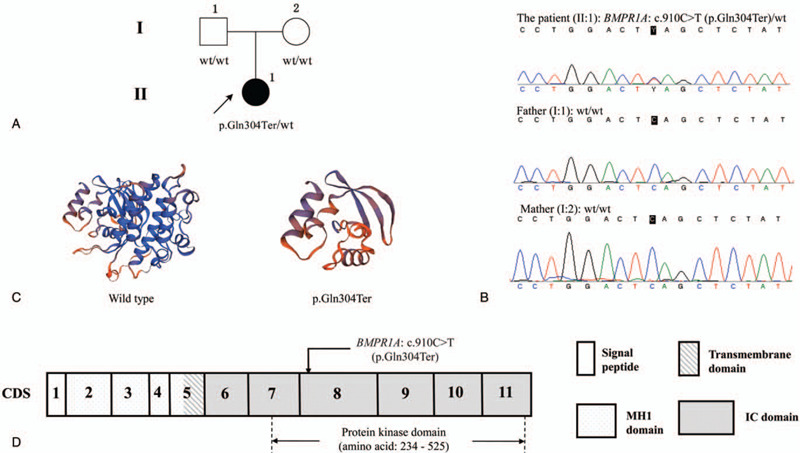
A novel de novo causative variant (*BMPR1A*: c.910C>T [p.Gln304Ter]) was identified in this family. A, Pedigree analysis. B, Sanger sequencing of this family. C, Protein structure prediction of this variant using the Swiss-model tool. D, Gene structure of *BMPR1A* and the identified variant.

After data analysis of the NGS, we achieved an average output of 337,770 mapped reads and 94.74% on-target coverage. The mean depth was 271.2-folds and the mean uniformity of base coverage was 98.20%. A total of 179 genetic variants were initially identified in this patient. The most promising candidate variant was *BMPR1A*: c.910C>T (NM_004329.2). It is a nonsense variant at exon 10 of *BMPR1A* gene, leading to a premature stop codon at the position of 304th amino acid (p.Gln304Ter, NP_004320.2). Pedigree analysis demonstrated that the patient carried a heterozygous *BMPR1A*: c.910C>T (II: 1) but her unaffected parents did not carry it (II: 1 and I: 2), suggesting a de novo mutation in this family (Fig. 1A and B). Structure prediction revealed a lack of nearly half parts of the normal protein in the mutant product (Fig. 1, C and D). In view of the kinase domain of BMPR1A (amino acid 234–525), c.910C>T could result in a 75% loss of the protein kinase domain. In addition, this variant has neither been found in multiple population databases (ExAC, 1000GP, genomeAD, etc.), nor recorded in disease databases including Clinvar and HGMD. Several bioinformatics tools (MutationTaster, VarCards, etc.) consistently supported a damaging effect of this variant. Taken together, *BMPR1A*: c.910C>T (p.Gln304Ter) is considered as a novel de novo pathogenic variant causing JPS (Table 1).

**Table 1:** Classification of the candidate variant according to ACMG guidelines.

	*BMPR1A*: c.910 C>T (p.Gln304Ter)
Mutation type	Nonsense, leading to a premature termination codon for NMD (PVS1)
Pedigree analysis	Only detected in the patient but not in her unaffected parents (PS2)
Population data^∗^	Absent in multiple normal population databases, including ExAC, 1000GP, genomeAD, and dbSNP (PM2)
Computational evidences^†^	Multiple computational evidences (MutationTaster and VarCards) supported a deleterious effect on *BMPR1A* gene product including conservation, evolutionary and struction (PP3)
Conclusion	Pathogenic (1 PVS1 + 1 PS2)

1000GP = 1000 genomes project, ExAC = exome aggregation consortium, NMD = nonsense-mediated decay, PM = pathogenic moderate, PP = pathogenic supporting, PS = pathogenic strong, PVS = pathogenic very strong.

∗ ExAC, http://exac.broadinstitute.org/); 1000GP, http://www.internationalgenome.org/1000-genomes-browsers; genomeAD, https://gnomad.broadinstitute.org/; dbSNP, https://www.ncbi.nlm.nih.gov/snp/.

† MutationTaster, http://www.mutationtaster.org/; VarCards, http://159.226.67.237/sun/varcards/welcome.

Based on our results, we provided surveillance suggestions including endoscopic screening for this family according to latest guidelines about JPS.^[11,12]^ Considering that each affected individual has a 50% chance to transfer the causative variant to the child, we also offered genetic suggestions to the patient. This family could benefit from timely surveillance and intervention.

## 3 Discussion

In this work, we reported a concealed case who came to hospital for fever and unexpectedly found to be JPS without previous medical history and JPS family history. By genetic testing and pedigree analysis, we identified a novel de novo pathogenic variant, *BMPR1A*: c.910C>T (p.Gln304Ter), in this Chinese JPS family, and further provided timely interventions and suggestions.

JPS is a one of hereditary GI cancer syndromes with variable clinical features. A clinical diagnosis of JPS is determined when a patient has any of the following condition: > 5 juvenile polyps of the colorectum; several juvenile polyps in the GI tract; or a family history of JPS and any number of juvenile polyps (National Comprehensive Cancer Network [NCCN] guidelines, https://www.nccn.org/professionals/physician_gls/default.aspx). However, Polyp burden of JPS differs by age at diagnosis and cell histology, and some carriers have adenomatous and/or sessile serrated polyps, leading to inconclusive clinical diagnosis. Genetic testing is a newly promising way for early diagnosis of JPS, especially for those affected individuals with no family history of JPS. Identification of a heterozygous germline pathogenic variant in *BMPR1A* or *SMAD4* could provide a definite diagnosis of JPS. Genetic testing can also determine affected individuals before occurrence of serious outcome including cancer, promote them to take early and effective intervention. In this case, genetic testing by multigene panel is a good way for early detection and definite diagnosis, both for the affected or at-risk individuals with hereditary GI diseases including JPS.^[11]^ Just like in our case, the patient was fortuitously identified as JPS during her treatment for fever and infection. She reported no conscious symptom, but colonoscopy found more than 20 polyps in the ileocecum and rectum. Some resected polyps had progressed to precancerous lesions such as high-grade dysplasia and adenoma. The genetic testing by a multigene panel helped the patient identify the disease cause and get timely interventions and genetic suggestions.

To better understand the association between genotype and phenotype, we further summarized latest evidence to delineate features of *BMPR1A*-related disorders, and compared the difference between mutation carriers of *SMAD4* and *BMPR1A*. Table 2 summarizes the extended phenotypic spectrum beyond JPS attributed to pathogenic or likely pathogenic variants of *BMPR1A*. *BMPR1A* encodes a crucial subunit of serine/threonine kinase receptor complex in the TGF-β pathway, which was reported as the second causative gene of JPS by Howe et al^[13]^ in 2001. The most common extra-JPS phenotype was hereditary mixed polyposis syndrome (HMPS), which probably resulted from large genomic deletion, small deletion, or nonsense variants of *BMPR1A*.^[14–16]^ It was reported that germline disease-causing variants of *BMPR1A* were detected in 4/8 (50%) of the HMPS families. Extra-colonic tumors could occur in some *BMPR1A*-related HMPS patients, such as thyroid cancer and Wilms tumor.^[15]^ Other polyposis including unexplained hereditary adenomatous polyposis > 100 polyps, unexplained adenomatous polyposis with unknown inheritance, and mixed polyposis, were also reported to be associated with frameshift, splicing, or missense variants of *BMPR1A*.^[17]^ In addition, large/small deletion of *BMPR1A* possibly led to familial colorectal cancer type X and sporadic early-onset CRC.^[18,19]^ Recently, pathogenic missense variants of *BMPR1A* were suggested to involve in the development of superior coloboma and congenital heart diseases (CHD).^[20,21]^ In addition, *BMPR1A* variants were also shown to be related to primary ovarian insufficiency,^[22]^ atrioventricular septal defect,^[23]^ and left ventricular noncompaction,^[24]^ but these identified variants were almost rare missense variants with unknown clinical significance. More studies are required to further validate the results.

**Table 2:** Extended phenotypic spectrum beyond JPS attributed to pathogenic or likely pathogenic variants of *BMPR1A*.

Disease phenotype	Disease description	Mean/median age at diagnosis	Detection method	Mutation type^∗^	Mutation proportion	Other conditions	Reference
HMPS	Polyps are mixed adenomatous, hyperplastic, or atypical juvenile histology, probably eventually leading to CRC.	33–61 y	Sequence analysis and MLPA	Large genomic deletion, small deletion, or nonsense	4/8 families (50%)	CRC, thyroid cancer and wilms tumor	^[14–16]^
Other polyposis	Including unexplained hereditary adenomatous polyposis > 100 polyps, unexplained adenomatous polyposis with unknown inheritance, and mixed polyposis	50 y	NGS and CNV analysis	Frameshift, splicing or missense	3/23 patients (13%)	A patient with an atypical polyposis carrying both *BMPR1A* and *CHEK2* variants.	^[17]^
FCCTX	It is a type of hereditary nonpolyposis colorectal cancer based on Amsterdam criteria for Lynch syndrome, which has no germline mutation in MMR, and the tumors are microsatellite stable.	54.6 y	Genetic linkage analysis and mutation analysis	Small deletion	2/18 families (11%)	CRC, lymphoma, scirrhous carcinoma in breast, etc.	^[18]^
Sporadic early-onset CRC with MMR proficiency	Early-onset (<50 y) CRC without MMR deficiency, no previous polyposis and no CRC family history	NA	Affymetrix 6.0 array	Large genomic deletion (a 7.326-Mb heterozygous deletion in the 10q22-q23 region including *BMPR1A*)	NA	NA	^[19]^
Superior coloboma	A congenital ocular anomaly characterized by gaps in the tissues of the superior eye	9.5 y	NGS and functional experiment	Missense	1/5 patients (20%)	NA	^[20]^
CHD	A most common organ malformations in the newborns, including atrioventricular septal defects, an atrial septal defect, and a ventricular septal defect.	NA	Genome-wide human SNP Array 6.0 and functional experiment	Missense	12/19 members in one family	A single patient suffered from “severe malformations” before his death during the first days after birth. No extra-cardiac anomalies were reported in this family.	^[21]^

CHD = congenital heart diseases, CNV = copy number variation, FCCTX = familial colorectal cancer type X, HMPS = hereditary mixed polyposis syndrome, MLPA = multiplex ligation-dependent probe amplification, MMR = mismatch repair gene, MMR = mismatch repair gene, NA = not available, NGS = next-generation sequencing.

∗ All pathogenic or likely pathogenic variants above were heterozygous.

As another causative gene of JPS, *SMAD4* encodes a downstream effecter of *BMPR1A* in the TGF-β signaling pathway. Inactivation of either of *BMPR1A* and *SMAD4* is a crucial step in polyp development of JPS. They have many similarities including similar core pathways, inheritance model, and PV proportion, but also have several differences (Table 3). Pathogenic defect of *BMPR1A* or *SMAD4* results in nearly 30% of JPS cases respectively, and most defects are point mutation and indels. Many JPS patients are asymptomatic or have nonspecific symptoms including stomachache and anemia, especially for *BMPR1A*-related JPS. However, *SMAD4* mutation carriers often show characteristic features such as JPT/HHT syndrome. Besides JPS and GI cancers, *BMPR1A* is also associated with HMPS, polyposis, superior coloboma, and CHD, while *SMAD4* is also associated with HHT, Myhre syndrome, Loeys-Dietz syndrome, cholangiocarcinoma, and thoracic aortic aneurysms and aortic dissections. More details about the comparison of the 2 genes are shown in Table 3.

**Table 3:** Comparisons between features of BMPR1A and SMAD4^∗^.

	BMPR1A	SMAD4
Chromosome location	10q23.2	18q21.2
Common core pathway	TGF-β signaling pathway, BMP signaling pathway	
JPS		
Inheritance model	Autosomal dominant	
Median age at diagnosis	18.5 y	
Cumulative risks of CRC	38% to 68% (average age at diagnosis: 34–44 y)	
Cumulative risks of extra-colorectal cancer	21% of incidence of upper GI cancer including stomach, pancreas, and small bowel (average age at diagnosis: 54 y)	
Proportion of JPS attributed to PV	28%	27%
Proportion of PV by detected methods	69% to 85% by sequence analysis, and 15% by large deletion/duplication analysis.	83% by sequence analysis and 17% by large deletion/duplication analysis.
Clinical features	NO obvious symptoms; nonspecific symptoms including digestive symptoms and anemia could occur in some patients.	A higher frequency of gastric polyposis; and often accompanied by extra-GI symptoms such as HHT features (epistaxis, telangiectases, visceral AVM, etc.)
Related diseases beyond JPS	HMPS, polyposis, GI cancers, superior coloboma, and CHD	HHT, GI cancers, Myhre syndrome, Loeys-Dietz syndrome, cholangiocarcinoma, and TAAD
Surveillance	Complete blood count, colonoscopy, and upper endoscopy should begin at age of 15 y or at the occurrence of initial symptoms, whichever is earlier. Genetic testing should be offered to children at risk for carrying PV of SMAD4 before age 15 y, because the surveillance for HHT-associated findings should begin earlier than the surveillance for polyps.	

∗ Data were summarized from GeneReviews (https://www.ncbi.nlm.nih.gov/books/NBK1116/), Genetics Home Reference (https://ghr.nlm.nih.gov), and ACG Clinical Guideline.^[1]^

In summary, we presented a case who was unexpectedly diagnosed as JPS during the treatment for infection. After genetic testing, we identified a novel de novo pathogenic variant, *BMPR1A*: c.910C>T (p.Gln304Ter), in this Chinese JPS family, and summarized *BMPR1A*-related features based on the latest evidence. Our findings not only extend the mutation spectrum of *BMPR1A*, but also delineate characteristics of *BMPR1A* mutation carriers. Often-overlooked symptoms, variable features, and lack of family history probably lead to a great underestimation of the number of potential affected individuals with JPS. Routine physical examination is difficult for identification, but genetic testing could make it. In view of benefits from early detection, we strongly suggest performing genetic testing by a multigene panel in individuals who have suspected symptoms of polyposis, especially in those with family history.

## Author contributions

**Conceptualization:** Fang Xiao and Juan Xiao

**Data curation:** Na Shen and Xiong Wang

**Methodology:** Xiong Wang and Yanjun Lu

**Supervision:** Juan Xiao

**Writing–original draft:** Na Shen

**Writing–review & editing:** Yanjun Lu, Fang Xiao and Juan Xiao

## Supplementary Material

Supplemental Digital Content
