# Supplementary material for: Importance of early detection of juvenile polyposis syndrome: A case report and literature review
Source: Medicine (Baltimore). 2020 Dec 11;99(50):e23494. doi: 10.1097/MD.0000000000023494 (PMC7738017; doi:10.1097/MD.0000000000023494)
Supplement: Supplemental Digital Content [file medi-99-e23494-s001.docx]

Supplementary Table S1. A 50-gene panel on hereditary gastrointestinal cancer syndromes

| **Type** | **Syndrome** | **Inheritance** | **Gene** |
| --- | --- | --- | --- |
| Hereditary nonpolyposis colorectal cancer | Lynch syndrome | AD | MLH1, MSH2, EPCAM, MSH6, PMS2, PMS1, MLH3, TGFBR2 |
| Hereditary adenomatous polyposis syndrome | Turcot’s syndrome | AD | APC, MLH1, PMS2 |
|  | Familial adenomatous polyposis (FAP) | AD | APC |
|  | MUTYH-associated polyposis (MAP) | AR | MUTYH |
| Hamartoma/Mixed/Hyperplastic polyposis syndrome | Constitutional mismatch repair deficiency syndrome | AR | MLH1, MSH2, MSH6, PMS2, EPCAM |
|  | Peutz-Jeghers syndrome (PJS) | AD | STK11 |
|  | Juvenile polyposis syndrome (JPS) | AD | SMAD4, BMPR1A |
|  | Sessile serrated polyposis cancer syndrome | AD | RNF43 |
|  | Cowden syndrome | AD | PTEN |
|  | Hereditary mixed polyposis syndrome | AD | GREM1 |
|  | MSH3-associated polyposis | AR | MSH3 |
|  | NTHL1-associated polyposis | AR | NTHL1 |
|  | hereditary hemorrhagic telangiectasia(HHT) | AD | ENG |
|  | Birt-Hogg-Dube Syndrome(BHDS) | AD | FLCN |
|  | Li-Fraumeni Syndrome | AD | TP53, CHEK2 |
|  | oligodontia-colorectal cancer syndrome | AD | AXIN2 |
|  | Polymerase proofreading-associated polyposis (PPAP) | AD | POLE, POLD1 |
| Hereditary gastric tumor | Hereditary diffuse gastric cancer (HDGC) | AD | CDH1, IL1B, IL1RN, KRAS |
|  | Familial gastrointestinal stromal tumor (GIST) | AD | KIT, PDGFRA, SDHC |
| Hereditary pancreatic cancer syndrome | Hereditary pancreatic cancer predisposition syndromes | - | BRCA1, BRCA2, STK11, ATM, PALB2, CDK4, CDKN2A, PRSS1, CFTR, SPINK1, CASR, CTRC, MLH1,MSH2, MSH6, PMS2, EPCAM, TP53, APC |
| Other hereditary diseases that significantly increase the risk of gastrointestinal cancers | hereditary breast and ovarian cancer (HBOC) | AD | BRCA1, BRCA2 |
|  | Cystic fibrosis (CF) | AR | CFTR |
|  | Familial atypical multiple mole melanoma | AD | CDKN2A |
|  | Hereditary pancreatitis | AD | PRSS1, SPINK1, CASR, CTRC |
|  | Ataxia-telangiectasia syndrome (A-T) | AR | ATM |
|  | carney syndrome (CNC) | AD | PRKAR1A |
|  | Tuberous sclerosis complex (TSC) | AD | TSC1, TSC2, IFNG |
|  | Neurofibromatosis (NF) | AD | NF1, NF2 |
|  | Bloom syndrome | AR | BLM |

Abbreviation: AD, autosomal dominant inheritance; AR, autosomal recessive inheritance.
